# Rice Plant Counting, Locating, and Sizing Method Based on High-Throughput UAV RGB Images

**DOI:** 10.34133/plantphenomics.0020

**Published:** 2023-01-30

**Authors:** Xiaodong Bai, Pichao Liu, Zhiguo Cao, Hao Lu, Haipeng Xiong, Aiping Yang, Zhe Cai, Jianjun Wang, Jianguo Yao

**Affiliations:** ^1^ School of Computer Science and Technology, Hainan University, Haikou 570228, China.; ^2^ School of Telecommunication and Information Engineering, Nanjing University of Posts and Telecommunications, Nanjing 210003, China.; ^3^ School of Artificial Intelligence and Automation, Huazhong University of Science and Technology, Wuhan 430074, China.; ^4^ School of Computing, National University of Singapore, Singapore 119077, Singapore.; ^5^ Agricultural Meteorological Center, Jiangxi Meteorological Bureau, Nanchang 330045, China.

## Abstract

Rice plant counting is crucial for many applications in rice production, such as yield estimation, growth diagnosis, disaster loss assessment, etc. Currently, rice counting still heavily relies on tedious and time-consuming manual operation. To alleviate the workload of rice counting, we employed an UAV (unmanned aerial vehicle) to collect the RGB images of the paddy field. Then, we proposed a new rice plant counting, locating, and sizing method (RiceNet), which consists of one feature extractor frontend and 3 feature decoder modules, namely, density map estimator, plant location detector, and plant size estimator. In RiceNet, rice plant attention mechanism and positive–negative loss are designed to improve the ability to distinguish plants from background and the quality of the estimated density maps. To verify the validity of our method, we propose a new UAV-based rice counting dataset, which contains 355 images and 257,793 manual labeled points. Experiment results show that the mean absolute error and root mean square error of the proposed RiceNet are 8.6 and 11.2, respectively. Moreover, we validated the performance of our method with two other popular crop datasets. On these three datasets, our method significantly outperforms state-of-the-art methods. Results suggest that RiceNet can accurately and efficiently estimate the number of rice plants and replace the traditional manual method.

## Introduction

Rice is one of the three major food crops in the world. Approximately 162 million hectares of land are used for rice cultivation worldwide, with more than 755 million tons of paddy rice produced [1]. Rice plant counting in paddy field is a fundamental work and has a wide range of applications in rice production. In cultivation management, rice counting can be applied to choose a more effective planting density to improve the nutrition competition between crops and weeds [2,3]. In rice growth observation, plant counting can be used for the disaster assessment caused by typhoons and floods [4,5]. Moreover, in crop phenotype analysis, the number of survival rice plants is one of the key metrics in rice breeding. With plant counting, breeders are able selectively to choose parents to hybridize to cultivate the next generation of excellent rice varieties [6]. At present, rice plant counting in China still heavily relies on manual sampling and statistics. The manual counting method has many disadvantages. Initially, plant counting in the field is labor-intensive and cumbersome. Furthermore, because of time and cost constraints, plant counts can only be performed in a small area in paddy field, making the results unrepresentative. In addition, human error often occurs during tedious manual counting. Finally, the manual observation in field tends to cause irreversible damage to rice. In this paper, we mainly focus on the accurate count of rice plants in paddy field with high-throughput unmanned aerial vehicle (UAV) images. Moreover, we also try to provide higher-level semantic information (plant location and size) that can contribute to downstream researches.

Many counting related techniques have been presented in agricultural research. Decision tree classifier and geometric descriptors were adopted to quantify early-season stand counts in corn [7]. Root nodule counting is utilized to indicate soybean health [8]. The pest population was counted to reduce the risk of crop exposure to pests and diseases [9]. Wheat ear counting approaches were proposed to estimate ear density under field conditions with zenithal color digital images [10] or thermal images [11]. The panicle numbers of pot-grown rice were determined by multiangle imaging and image segmentation [12]. Rice heading stage was automatic observation under field conditions by multiclassifier-cascade-based rice spike detection procedures [13]. An indoor grain image acquisition system and grain counting algorithm were proposed for touching hybrid rice [14]. A Faster RCNN (Faster Region Convolutional Neural Network) detection method is utilized to detect wheat ears in an image [15]. Maize tassel counting method TasselNet was proposed by the means of local patch regression [16]. Liu et al. [5] used the image acquisition camera fixed in the field to obtain the rice image in a fixed area and realized the calculation of the rice planting density. Although many excellent approaches have been presented on corn stand [7], root nodule [8], pest [9], wheat ear [11], rice spike [13], etc. counting tasks, none of them can be used to accurate rice plant counting in paddy field. First, unlike the low-cost RGB camera that we use, previous researches may need to apply expensive infrared camera [11]. Second, some previous researches require specific imaging conditions, such as controlled illumination [9], multiangle imaging [14], and near range imaging at a fixed position [5,8,15]. Third, many traditional technologies are utilized in previous methods, such as the color threshold segmentation [12], watershed operation [14], and manual character classification [7,9]. It will make their methods unable to adapt to the light variation in rice field. Last but not least, the counting objects in previous researches are different from us [10,13,16]. Obviously, different image objects and datasets have their own characteristics. For the counting methods well-designed with other datasets, Their performance cannot be guaranteed in the tasks of rice plant counting. Indeed, it is still an open problem to count rice plants in paddy field accurately and efficiently.

Besides agricultural scenes, many achievements have been acquired in crowd counting [17–19], vehicle counting [20], cell counting [21,22], etc. Since crowd counting is the biggest and most successful branch in counting research, we mainly review the researches on crowd counting in this paper. Toward crowd counting, early methods were mainly based on detection [23], which applied low-level feature descriptors [24] and a sliding-window-based detection to obtain the object number. Essentially, these detection-based methods are usually based on classification, which requires a large number of manual bounding box annotations [25,26]. Of course, it is very time-consuming to prepare a large enough dataset for detection-based counting methods. However, severe occlusion, high congestion, and adhesion can make these methods perform poorly. To conquer the above difficulties, later researchers proposed regression-based methods that regress the object density map with the input image and then integrate the density map to obtain the count [27]. Nowadays, deep learning has brought unprecedented success to the research of crowd counting. Zhang et al. [18] are the first to apply deep learning to simultaneously estimate object density maps and object counts. Since then, deep learning has become the mainstream method [5,28–32]. The great breakthrough in crowd counting has important reference significance for the research of crop plant counting. However, there are still many shortcomings in the current deep learning counting methods. To begin with, the influence of irrelevant image background is still not completely solved, which will result in many false positives and a decline in counting accuracy [30,32,33]. In addition, many deep-learning-based counting networks need to apply complex image augmentation to ensure their performance [31,34–36]. Furthermore, the existing deep learning methods are mainly focused on counting. There are still many other applications that have not been excavated [5,28,29,37].

With the popularity of UAVs, the collection of high-throughput crop images has become more accessible and affordable in crop phenotype research [38,39]. Using the acquired rice images in paddy field, we found that we can not only obtain the number of rice plants but also realize their location and size estimation, which can be beneficial to downstream crop phenotype analysis. However, plant location and size estimation have not received enough attention recently. On the basis of the above discussions, the application of UAVs to complete field rice image acquisition and then design new deep learning networks to achieve automatic rice plant counting, locating, and sizing has far-reaching research significance.

In this paper, we present a new deep learning network RiceNet that can realize rice plant counting, locating, and sizing in paddy field with high-throughput RGB images from UAV. After considering the aforementioned shortcomings in current deep-learning-based counting methods, we made the following improvements in the design of RiceNet network: (1) To more effectively characterize the rice plants in high-throughput field images, multiscale features of different semantic information levels are carefully abstracted and incorporated in RiceNet. (2) To guide network to pay attention to more useful information in forward propagation, plant attention mechanism is presented and adopted in RiceNet. (3) Besides plant counting, RiceNet can also provide the plant location and size information by the presented plant location detector (PLD) and plant size estimator (PSE) modules. Those plant location and size information can contribute to downstream crop phenotype researches. In general, sufficient number of manually labeled bounding boxes is required for deep learning network to realize the locating and sizing of rice [40]. Of course, it is time-consuming and laborious to manually label thousands of rice plants with bounding boxes on a high-throughput rice image. On the basis of the reasonable assumption that rice plants are evenly distributed in the image, we initialize pseudo-rice plant bounding boxes from the point-level supervisory. Then, we leverage those pseudo-bounding boxes to guide the regression of rice sizes via an L1 loss. With the same assumption in the obtained high-quality estimated density map, a local nonmaximum suppression (LNMS) is utilized to the estimated density map to get the plant location. Finally, by fusing the obtained location results with the size information given by PSE, RiceNet brings an efficient estimation of the plant size. (4) A new positive–negative loss is proposed in RiceNet, which can be combined with *L_mse_* and *L_bce_* to enable network parameters to iterate accurately. The utilization of (1), (2), and (4) allows RiceNet to distinguish the rice plants in image and suppress the false positives in background more effectively. All the source code of RiceNet is available at https://github.com/xdbai-source/Rice-Plant-Counting.

In the current counting research, most count datasets are aimed at the crowd [19,30], cells [22], spikes [41], or flowers [42]. There is no UAV-based field rice counting dataset right now. In our research, we adopt low-altitude UAV to acquire RGB images of two paddy field from 2018 to 2020. Then, we propose a new UAV-based rice counting (URC) dataset that contains 355 image and 257,793 manual labeled points of rice plants. According to the current literature survey, we are the first to propose a rice plant counting dataset with UAV images, and the first to propose an efficient approach that simultaneously realizes the counting, locating, and sizing of rice. In experiments, our proposed method achieves state of the art on 3 datasets in terms of counting accuracy. In summary, our main contributions in this paper are as follows:

1. We present a new UAV-based high-throughput rice plant dataset (URC dataset) for the counting research of field rice plants. According to the current literature survey, this is the first rice plant counting dataset with UAV RGB images.

2. We propose a new network RiceNet that can achieve automatic, contactless, and accurate counting of rice plants in a large paddy field with UAV RGB imagery. In RiceNet, multiscale feature fusion, plant attention mechanism, and positive–negative loss are presented and adopted to suppress false positives from the image background to generate high-quality density maps. Experiments show that the proposed RiceNet outperforms the state of the arts on our URC dataset and 2 other popular datasets.

3. With the designed PLD and PSE modules in RiceNet, our method also brings higher-level semantic information such as plant location and size. This high-level semantic information is of great significance to downstream phenotyping research tasks.

## Materials and Methods

### UAV-based crop RGB image acquisition

**Fig. 1.**
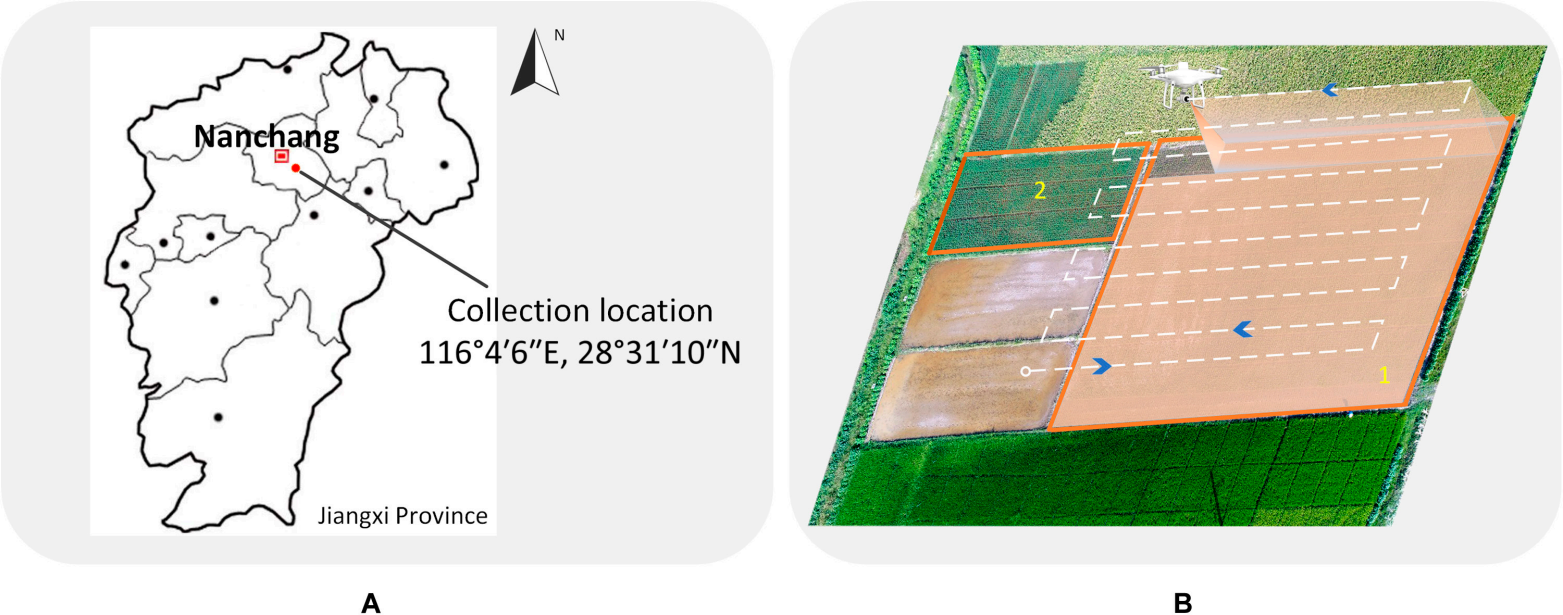
Image collection process of rice field based on quadcopter UAV. (A) gives the collection location. (B) shows the image collection process in field 1 and field 2, respectively.

The rice images used in the experiment were collected in Nanchang City, Jiangxi Province, China. Its location is shown in Fig. 1A. The rice variety used for observation is indica-type rice. As shown in Fig. 1B, two rice fields were applied to collect the images by a quadcopter UAV (DJI Phantom 4 Advanced) and DJI GS Pro ground station. DJI GS Pro was utilized to automatically generate efficient flight paths and waypoints to complete the aerial photographing missions of the specified paddy fields. In DJI GS Pro, the flight altitude was fixed at 7 m above the ground. The RGB camera on the drone applied the vertical downward shooting. To prevent image blur, the image acquisition method of hovering shooting was adopted at each waypoint. The front and side overlap ratios were set to 80% and 70%, respectively. In the first image acquisition mission, we have to create a new UAV flight plan and set all flight parameters in GS Pro carefully. In the following missions, we only need to open GS Pro and perform the above flight plan again. Rice images were collected every three days when the rice was between the tillering and jointing development stages from 2018 to 2020. For rainy and windy days when the UAV cannot take off, image acquisition will be postponed by one day. In this way, we collected an average of 25 UAV RGB image sequences per year. The original images with a resolution of 5,472 × 3,648 were captured and saved in the UAV SD (Security Digital) card in JPG format. After each flight mission, rice images were exported and stored in the data server.

### UAV-based rice counting dataset

**Fig. 2.**
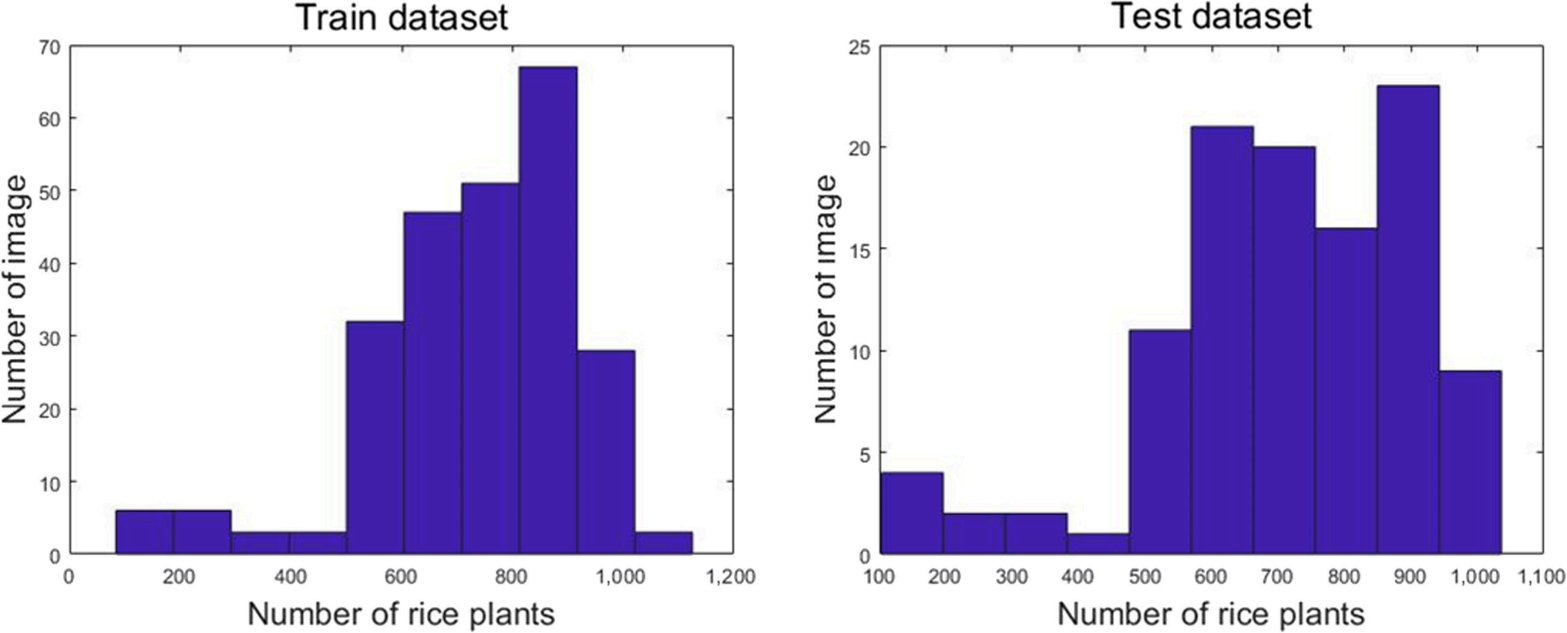
Statistical distribution of the URC dataset.

According to our existing literature survey, there is still no public UAV-based rice plant counting dataset. To realize the research of rice plant counting in a large paddy field, we present a new URC dataset in this paper. URC dataset contains 355 original high-throughput rice images (size of 5,472 × 3,648), which collected between 2018 and 2019. Following the standard practice [41], the center positions of all plants in each image in the URC dataset are manually annotated with dots. Compared with box labeling, point labeling is a more much reasonable and feasible way to represent so many rice plants in UAV images. Specially, we wrote a MATLAB R2018 script to accurately and conveniently label the center of each plant. Afterward, those images were downsampled into 0.25× of the original resolution to facilitate network training. Last, we got a dataset with 355 images (size of 1,368 × 912) and 257,793 manually labeled points. Among them, 246 images were randomly selected and used as training images and the remaining 109 images as test images. Each image contains rice plants ranging from 84 to 1,125, with an average of 726 plants per image. In Fig. 2, we provide the rice plant histograms of the images in URC dataset.

**Fig. 3.**
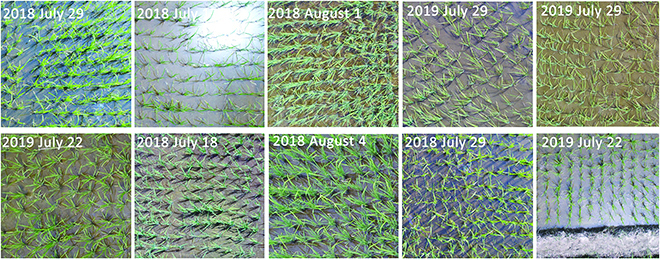
Examples of image acquisition from different dates.

**Fig. 4.**
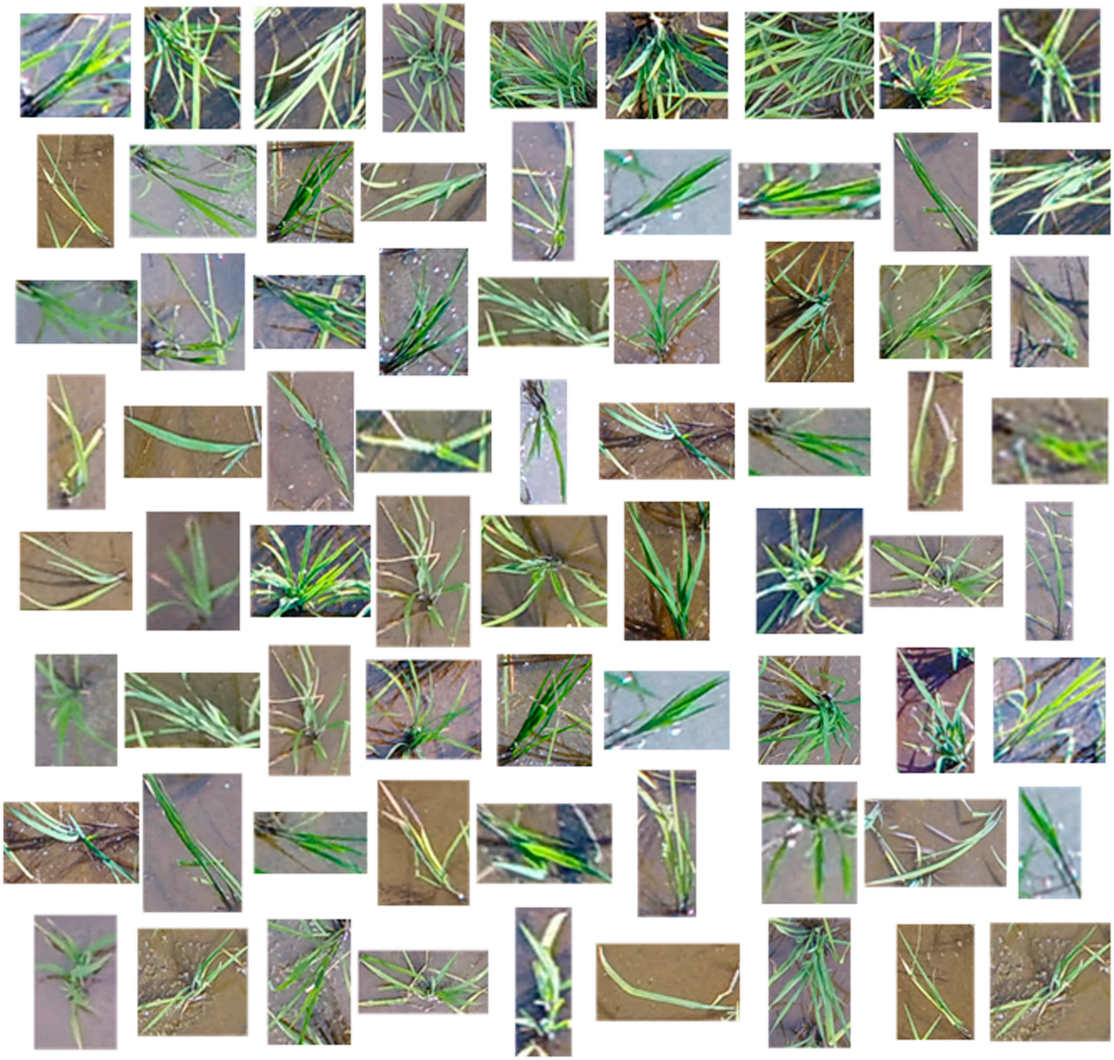
Diversity of plant morphology in URC dataset.

The diversity of the rice images collected by UAV in the URC dataset is well illustrated in Fig. 3. For the convenience of demonstration, the image blocks of ten original UAV images are given in Fig. 3. As can be seen in Fig. 3, our dataset contains rice images taken under many lighting and weather conditions. Our URC dataset is not a single scene, and it is very representative. Moreover, we extracted some individual rice plants from the images in the URC dataset, as shown in Fig. 4. Figure 4 shows the richness of rice plant traits in aspect ratio, color, orientation, size, etc. As shown in Figs. 3 and 4, URC dataset is a quite challenging dataset for rice plant counting.

### Network architecture

**Fig. 5.**
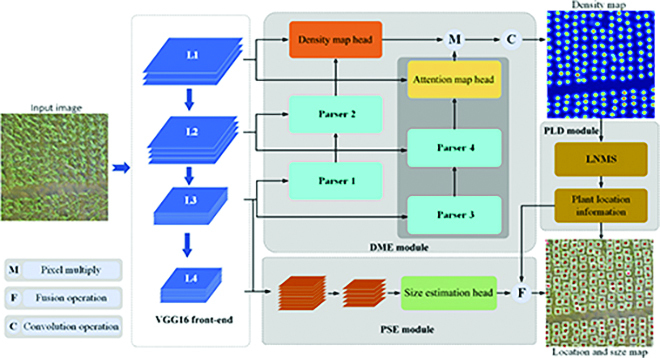
The overall architecture of the proposed RiceNet. An input image is fed into the feature extractor to generate four feature maps containing different semantic levels. The DME combined with plant attention mechanism to leverage multiple feature maps to generate a high-quality density map. The PLD and PSE are employed to output higher-level semantic information such as size and location.

In this section, we will introduce our network architecture. As illustrated in Fig. 5, RiceNet consists of a feature extractor and three designed network decoder modules, namely, density map estimator (DME), PSE, and PLD. In RiceNet, the first 13 layers of vgg16_bn [43] are used as the feature extractor. As shown in Fig. 5, an input image is first fed into the feature extractor, and then multilayer feature maps with different semantic levels are extracted. Next, the DME module adopts splicing and upsampling operations to fuse the multilayer features to generate high-quality estimated density map. Rice plant attention mechanism is added in the DME to improve the network’s ability to distinguish plants from background. Furthermore, RiceNet introduces the PLD module with LNMS to extract location information from the estimated density map and the PSE module to estimate the size information of the rice plants. Therefore, RiceNet can realize not only rice counting but also location and size estimation of the rice plants in UAV images. In the following subsections, we will describe each module in detail.

### Density map estimator

**Fig. 6.**
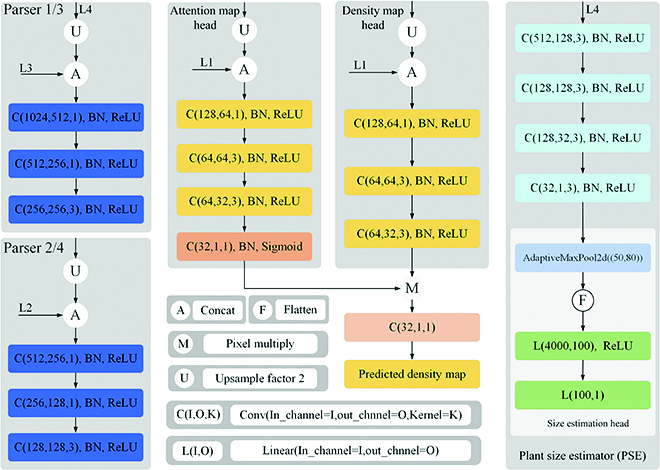
Module structure diagram of RiceNet. Parser 1/3 and Parser 2/4 respectively integrate feature maps of different levels. Attention map head and density map head are used to generate PAM and IDM, respectively. We can see that PSE is a lightweight module that realizes the size prediction.

We use the first 13 convolution layers in VGG16_bn [43] as the feature extractor of RiceNet. By the feature extractor, 4 multiscale features L1, L2, L3, and L4 were extracted from an input image. The downsampling rates of those feature levels are 1/2, 1/4, 1/8, and 1/16, respectively. Next, we introduce the structure of the designed DME module. As shown in Fig. 5, Parser 1 merges the feature maps of L4 and L3, and Parser 2 merges the output of Parser 1 and the L2 feature map. Then, a density map head combines the output of Parser 2 and L1 to get the initial estimated density map. Specifically, the structures of Parser 1 and Parser 2 are given in Fig. 6. In Parser 1, L4 is upsampled to the same size as L3 and merged with L3. Next, several convolutional layers are employed to get the output. Parser 2 upsamples the output of Parser 1 and merges it with L2. Similar to Parser 1, it is also fed into several convolutional layers before output. Finally, the density map head fuses L1 and the output of Parser 2 to obtain the initial density map (IDM). The resolution of IDM is 1/2 of the original input image.

To further suppress the interference of image background and improve the accuracy of the IDM, plant attention mechanism is presented and adopted in DME module as shown in Fig. 5. The plant attention mechanism is implemented by Parser 3, Parser 4, and attention map head. Their detailed network structures were given in Fig. 6. Similar to Parser 1 and Parser 2, Parser 3 and Parser 4 fuse the L4, L3, and L2 feature maps and then transfer its output into the attention map head. Attention map head merges the L1 with the output of Parser 4 and then followed by several convolutional layers. Finally, attention map head outputs a plant attention map (PAM), which is 1/2 size of the input image and used to refine IDM into a higher-quality density map. We can see that Parse 1 and Parse 3 share the same network design, and Parse 2 and Parse 4 have the same network design, which can reduce the complexity and difficulty of network model realization.

PAM will assist the IDM to distinguish the rice plants from the background and noise. In detail, element-wise multiplication and a convolution operation are applied on PAM and IDM to generate refined final density maps (FDMs) as Eq. 1:

$$\mathrm{FDM}=\text{Conv}\left(\mathrm{PAM}⊙\mathrm{IDM}\right)$$ (1)

where ⊙ means Hadamard product and *Conv* represents convolution operation.

Now, we describe how to generate a ground-truth density map *D^gt^*(*x*), which is used for network training. If there is a plant at pixel position *x_i_*, then we can represent it as a delta function *δ*(*x* − *x_i_*). Inspired by Lempitsky and Zisserman [27], we further convolve the above function with a Gaussian kernel *G_σ_*. Thus, suppose that there are *N* rice plants in an image, and ground-truth density map *D^gt^*(*x*) can be given as:

$$D^{\mathrm{gt}}\left(x\right)=\sum_{i=1}^{N}\delta \left(x-x_{i}\right)*G_{\sigma }\left(x\right)$$ (2)

Next, we also introduce the generation method of the ground truth of the PAM. For the generated ground-truth density map *D^gt^*(*x*), we believe that the nonzero pixel value region represents the existence probability of the plants. That is to say, the region with zero pixel values in *D^gt^*(*x*) is the image background. Therefore, the ground truth of the PAM can be written as:

$$\forall x_{i}\in D^{\mathrm{gt}}\left(x\right),G^{\mathrm{gt}}\left(x_{i}\right)=\{\begin{array}{ll} 1, & \text{if}\;x_{i}>0.001\\ 0, & \text{otherwise} \end{array}$$ (3)

### Plant location detector

**Fig. 7.**
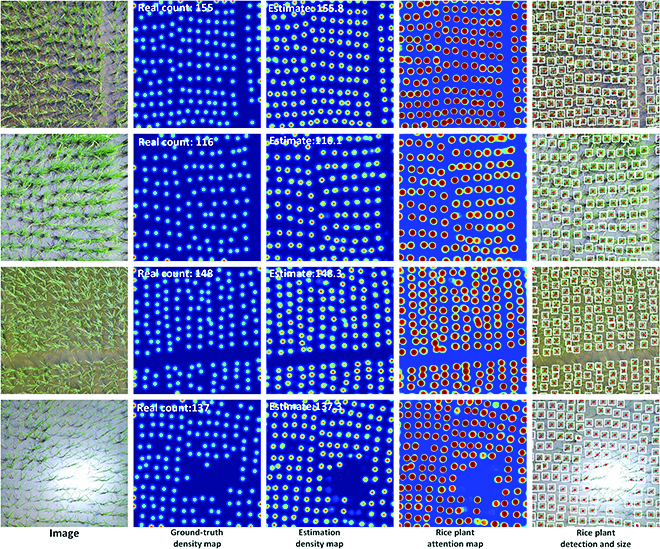
Visualization of the counting, locating, and sizing results in the URC dataset. The first column is the four test images, the second column is the ground-truth density maps, the third column is the estimated density maps, the fourth column is the PAMs, and the fifth column is the plant location and size prediction results.

In the third column of Fig. 7, we can see that the density distribution of our estimated the FDM is almost uniformly distributed and independent of each other. Naturally, each peak position on the FDM can be considered as the center position of a plant. Therefore, with a given estimated FDM, the locations of the plants can be obtained through the LNMS. First, we generate a pseudo-size for each image as its ground-truth size with the previous manual point annotations. If there is a plant at pixel position *x_j_*, following Shi et al. [44], then we first calculate the initial object size of point *x_j_* according to the distances to its *K*-nearest points, and then the pseudo-average size *D_mean_* of an image is calculated using Eqs. 4 and 5.

$${\bar{x}}_{j}=\frac{1}{K}\sum_{k=1}^{K}\beta x_{j,k}$$ (4)

$$D_{\text{mean}}=\frac{1}{M}\sum_{j=1}^{M}{\bar{x}}_{j}$$ (5)

where *x_j,k_* is the distance between point *x_j_* and its *k*th nearest neighbor. ${\bar{x}}_{j}$ represents the initial object size of *x_j_*. β is a scalar and generally can be set to 0.8. *M* is the number of the plants. Second, following Zhang et al. [30], we adopt max pooling to obtain local maximum mask map, where the max pooling kernel size is set to *D_mean_* × 0.3. Third, the local maximum mask map and the density map are multiplied to obtain all local maxima. However, those local maximum points may even contain some false positives from the background. According to the high-quality FDM generated by the DME module, we observe that the values of those false positives are much smaller than the true positives from the plants. It means that those positions with very small local maxima may originate from the image background. Finally, given an estimated FDM and its maximum pixel value *M*, an adaptive threshold *M* × 0.3 can be used to filter out the false positives. In particular, if the maximum value *M* is less than a small value (set to 0.01), then it means that there are no plants in the input image. The main flow of the PLD module to obtain the location information is shown in Algorithm 1.

**Table:** 

**Algorithm 1.** LNMS algorithm.
**Input:** FDM - Predicted FDM of a given image I; *D_mean_* - Pseudo-average size of plant in I. **Output:** The coordinates of the plant 1: **Function** Extract coordinates(*FDM,* *D_mean_*): 2: *Max_mask = max_pooling(FDM, kernel_size = (D_mean_* *0.3, *D_mean_* *0.3)) 3: *Max_mask = (Max_mask = FDM)* 4: *Max_all = Max_mask × FDM* 5: *adaptive threshold = max(Max_all)×0.3* 6: **if** *max(Max_all)<0.01* **then** 7: *coordinates = None* 8: **else** 9: *Max_all[Max_all > adaptive threshold] = 1* 10: *Max_all[Max_all < 1] = 0* 11: *coordinates = nonzero(Max_all)* 12: **end if** 13: **return** *coordinates* 14: **end function**

### Plant size estimator

Let ${\left\{d_{j}\right\}}_{j=1}^{M}$ represent the coordinates generated by Algorithm 1, where *d_j_* = (*x_j_*,*y_j_*) is the two-dimensional coordinates of the center of the *j*th estimated plant in an image, and *M* is the total number of coordinates. Then, we can estimate a size value for each plant through the *K*-nearest neighbors algorithm.

$${\bar{d}}_{j}=\frac{1}{K}\sum_{k=1}^{K}\beta \sqrt{{\left(d_{j}-d_{k}\right)}^{2}}$$ (6)

where ${\bar{d}}_{j}$ is the initial object size of ***d**_j_*, *d_k_* is the *k*th point closest to *d_j_***,** the value of *K* is 3, and *β* is a scalar. However, for the plants in sparse region, their sizes obtained by the Eq. 6 will be too large. Therefore, we design a size estimate head structure in our research to regress a size limitation of the plants in the input image. The detailed network structure of the PSE module is given in Fig. 6. In the PSE, L4 feature map is processed by several convolutional layers to further abstract high-level information. Afterward, an adaptive pooling, a flattening process, and 2 linear layers are applied to output the size limitation. We use L1 loss as the loss function of the PSE module and *D_mean_* (mentioned in Plant location detector section) as the regression target. Finally, the size estimation comes from the fusion of the PSE and the PLD outputs, as described in Fig. 5. If the output value of PSE module is *d_p_*, then we combine the output of PSE module to bring a more reasonable plant size *D_j_*, which can be written as:

$$D_{j}=\{\begin{array}{ll} d_{p}, & \text{if}\;d_{p}<{\bar{d}}_{j}\\ {\bar{d}}_{j}, & \text{otherwise} \end{array}$$ (7)

### Loss functions

In the training phase, we train the plant attention mechanism with a pixel-wise binary cross entropy loss between the network predicted PAM *M^p^* and its corresponding ground truth *G^gt^*, which can be given as:

$$L_{\mathrm{bce}}=-\frac{1}{N}\sum_{i=1}^{N}(G_{i}^{\mathrm{gt}}\cdot \log(M_{i}^{p})+(1-G_{i}^{\mathrm{gt}})\cdot \log(1-M_{i}^{p}))$$ (8)

where *N* is the batch size. Moreover, we train the DME module with mean squared error loss between the estimated density map *D^p^* and its corresponding ground-truth map *D^gt^* by:

$$L_{\mathrm{mse}}=\frac{1}{N}\sum_{i=1}^{N}{\left(D_{i}^{p}-D_{i}^{\mathrm{gt}}\right)}^{2}$$ (9)

To better suppress the false positives in background, we propose a positive–negative loss function. For the generated ground-truth density map *D^gt^*(*x*), we consider that the region with nonzero pixel value in ground-truth map is the positive region. Similarly, the region where the pixel value is zero is the negative region. The probabilities of having objects in the positive region and the negative region are *p* and 0, respectively. Correspondingly, the probabilities of being the background are 1 − *p* and 1, respectively. Let ${\left\{D^{\mathrm{gt}}(x_{m})\right\}}_{m=1}^{M}$ be a density map, where *x_m_* denotes a two-dimensional pixel location and *M* is the number of pixels in the density map *D^gt^*(*x*). So, we can get the object positive–negative probability map *P^O^*(*x_m_*) and background positive–negative probability map *P^B^*(*x_m_*) by:

$$\forall x_{m}\in D^{\mathrm{gt}}(x_{m}),P^{O}(x_{m})=\{\begin{array}{ll} p, & \text{if}\;x_{m}>0\\ 0, & \text{otherwise} \end{array}$$ (10)

$$\forall x_{m}\in D^{\mathrm{gt}}(x_{m}),P^{B}(x_{m})=\{\begin{array}{ll} 1-p, & \text{if}\;x_{m}>0\\ 1, & \text{otherwise} \end{array}$$ (11)

The expected counts for positive region and for the entire negative region are defined as

$$C_{O}=\sum_{m=1}^{M}P^{O}(x_{m})\cdot D^{p}(x_{m})$$ (12)

$$C_{B}=\sum_{m=1}^{M}P^{B}(x_{m})\cdot D^{p}(x_{m})$$ (13)

In this case, the summation over the whole density map *D^p^* consists of the object positive–negative counts *C^O^* and the background positive–negative count *C^B^*. Obviously, we would like the background positive–negative count to be zero, and the object positive–negative count is equal to the real count value *C^gt^*. Thus, we have the following positive–negative loss function,

$$L_{\mathrm{si}}=|(C^{\mathrm{gt}}-C_{O})+(0-C_{B})|$$ (14)

where *C^gt^* is total number of objects, so the total loss function is as follows:

$$L_{\mathrm{all}}=L_{\mathrm{mse}}+\lambda L_{\mathrm{bce}}+\gamma L_{\mathrm{si}}$$ (15)

### Evaluation metric

To assess the difference between the predicted counts and the ground-truth numbers, we applied the commonly used mean absolute error (MAE) and root mean square error (RMSE). The definitions of MAE and RMSE are as follows:

$$\mathrm{MAE}=\frac{1}{N}\sum_{i=1}^{N}|Z_{i}-{\hat{Z}}_{i}|$$ (16)

$$\text{RMSE}=\sqrt{\frac{1}{N}\sum_{i=1}^{N}{\left(Z_{i}-{\hat{Z}}_{i}\right)}^{2}}$$ (17)

where *Z_i_* is real number of rice plant in the *i*th image, ${\hat{Z}}_{i}$ is the estimated total number of plants in the *i*th image, and *N* is number of test image. The MAE indicates the accuracy of different approaches, while the RMSE reflects their robustness. A smaller value of MAE and RMSE indicates better network performance.

### Implementation details

In the experiment, to reduce computational consumption, the resolution of input images is downsampled into 1/4 of the original resolution of 5,472×3,648. Image blocks in 320 × 320 are randomly cropped from URC dataset images, and then they are randomly horizontal flipped with a probability of 0.5 and processed by a gamma contrast transform with a probability of 0.3 for data augmentation. Adam optimizer is utilized in the training process. The initial learning rate and batch size are set to 1 × 10^−4^ and 3, respectively. The hyperparameter λ and ϒ of the loss function are both set to 0.1. The value of *p* in the positive–negative loss function is set to 1. We first trained the DME module to output the estimated high-quality density maps. Afterward, the PSE module is trained with the parameter fine-tuning of the frontend feature extractor. Our experiment is implemented by the PyTorch framework and applied GPU NVIDIA RTX 3090 for acceleration.

## Results

### Experiment on the URC dataset

In this part, we compared our method with previous state of the arts and analyzed their results on the proposed URC dataset. Table 1 shows the performance of different methods on the URC dataset. The leftmost column is several advanced counting methods and our approach. The third and fourth columns are their MAE and RMSE results. As can be seen from Table 1, our method outperforms other methods by a large margin. The MAE and RMSE of the proposed method reach 8.6 and 11.2, respectively. RiceNet sets the new state of the art with clear advantages over other competitors on the URC dataset. What is striking about the figures in Table 1 is that compared with the TasselNetV2 and FIDTM, the MAE of our method is improved by 70.8% and 67.3%, respectively. We believe that the reason why RiceNet performed better than TasselNetV2 is that RiceNet adds new plant attention mechanism in our DME module and enhances parameter learning capability through new loss function. Compared with the crowd counting approaches including FIDTM, their networks mainly solve the challenges in head scale variation and density distribution imbalance. Obviously, this is not the case for the almost evenly distributed rice plants in paddy field. Thus, our method can focus more on improving the accuracy of plant counting.

**Table 1.** The performance of different methods on the URC dataset.

**Networks**	**Venue, year**	**MAE**	**RMSE**
MCNN [30]	CVPR, 2016	25.5	34.1
CSRNet [17]	CVPR, 2018	12.9	17.5
SANet [32]	ECCV, 2018	10.1	13.4
TasselNetV2 [41]	PLME 2019	29.5	39.4
FIDTM [45]	Arxiv, 2021	26.3	31.5
**RiceNet**	**This paper**	**8.6**	**11.2**

In the first and second columns of Fig. 7, we demonstrate four test images in the URC dataset and their ground-truth density map. The third column of Fig. 7 shows the estimated density map by RiceNet. We can see that the estimated high-quality density map in the third column is very close to the ground-truth density map. At the same time, we also give the visualization of the predicting rice PAM in the fourth column of Fig. 7. We can see that the plant attention mechanism in the model promotes the model to focus more on rice plants and plays an important role. To summary up, the adoption of new plant attention mechanism and new loss function (see Ablation experiment section) makes RiceNet more suitable for plant counting, which we believe is the reason why RiceNet performs better than other methods.

By comparing the second and third columns in the last row of Fig. 7, we can find that the estimated density map given by RiceNet is even slightly better than the ground-truth density map around the image region with strong sun reflection. This reflects that our network has a certain robustness to the sun reflection in paddy field. Actually, how to fully overcome the impact of the strong sun reflection is still an open problem that needs further research.

### Experiment on the MTC dataset

In addition, we also did comparison experiment on the MTC dataset. The MTC dataset is a maize tassels count dataset first introduced by Lu et al. [16]. It includes 361 images collected from four experimental fields across China between 2010 and 2015 with six maize cultivars. Maize tassels in the MTC dataset have very large scale and shape changes. The original resolution of those images is 3,648 × 2,736, 4,272 × 2,848, or 3,456 × 2,304. In this dataset, 186 images are randomly selected and used as the training set, and the remaining 175 images are used as the test set. The number of maize tassels in each image varies from 0 to around 100. In the experiment, the short sides of all images in the MTC are proportionally resized to 512 to speed up the calculation. Next, image blocks in the size of 256 × 256 are randomly cropped and used in the training. Other training details are consistent with the URC dataset. Table 2 shows the performance of different methods on the MTC dataset. As shown in Table 2, RiceNet achieves the best MAE (3.4) and RMSE (5.3), compared with other approaches. It is worth mentioning that our method achieves 81.0% improvement in MAE and 75.8% improvement in RMSE compared to the MCNN. Many images in the MTC dataset have very few maize tassels. This experiment shows that our network can also achieve good performance for the object sparse scenes. We believe that better performance in our method is due to its well object feature extraction and fusion ability between different levels of features. This indicates the superiority of the DME module in our method.

**Table 2.** Performance of different methods on the MTC dataset.

**Networks**	**Venue, year**	**MAE**	**RMSE**
MCNN [30]	CVPR, 2016	17.9	21.9
CSRNet [17]	CVPR, 2018	6.9	11.5
BCNet [46]	TCSVT, 2019	5.2	9.2
SFC^2^Net [5]	PLPH, 2020	5.0	9.4
TasselNet [16]	PLME, 2017	6.6	9.9
TasselNetV2 [41]	PLME, 2019	5.4	9.2
**RiceNet**	**This paper**	**3.4**	**5.3**

**Fig. 8.**
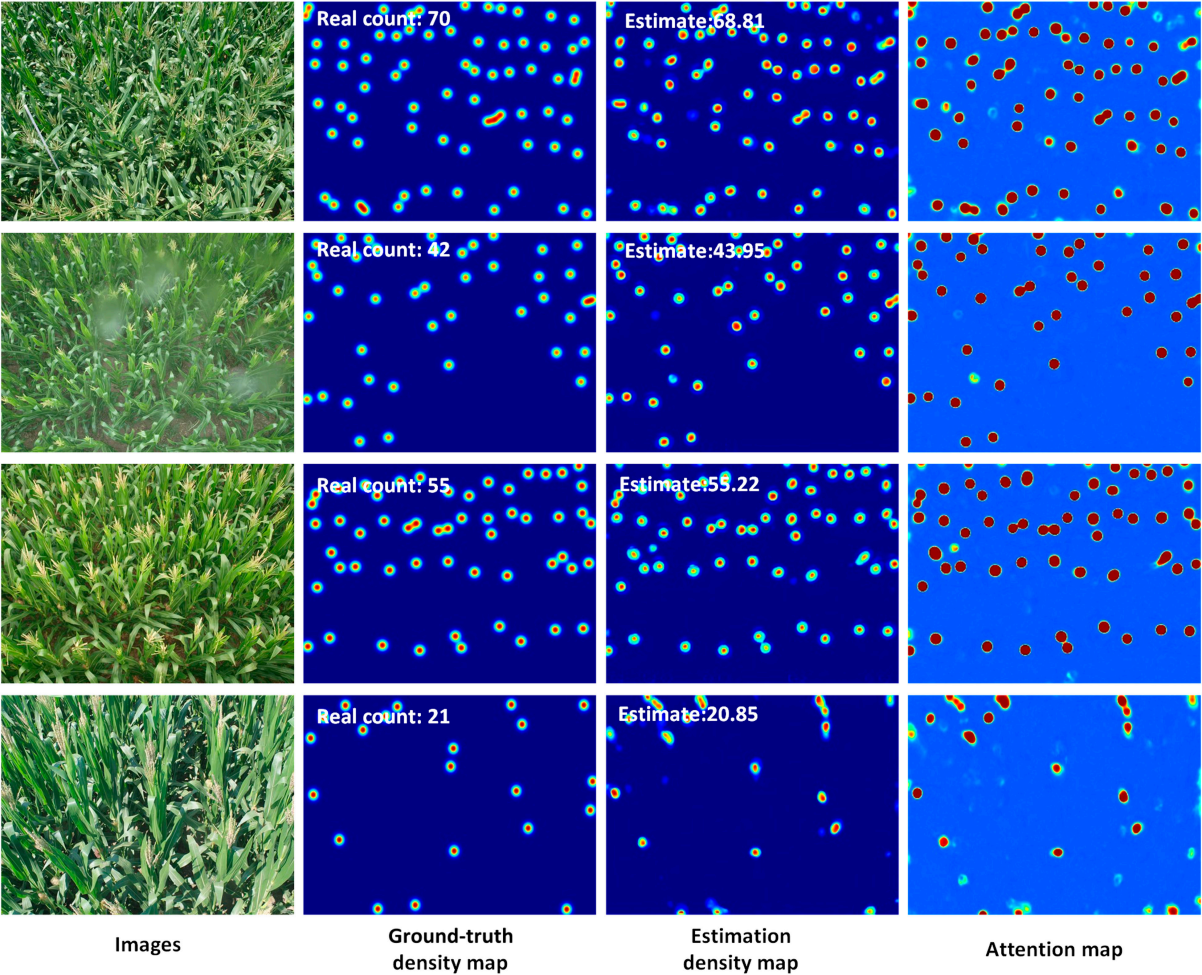
Visualization of the counting results in the MTC dataset. The first column is the 4 test images, the second column is the ground-truth density maps, the third column is the estimated density maps, and the fourth column is the PAMs.

In addition, some counting results in this dataset are demonstrated in Fig. 8. The third column of Fig. 8 shows that the estimated density map is similar to the ground-truth density map and the estimated counting result is close to the real value. We found that several maize tassels were lost in the network estimation density map. This phenomenon is mainly due to the shape and size of these maize tassels that are too special compared to others. As seen from the second row of Fig. 8, our network gives a high-quality estimation density map and counting result even when there are raindrops in the original RGB maize image. From another aspect, it shows the well performance of our proposed network. Briefly, Table 2 and Fig. 8 indicate that the proposed RiceNet can also achieve good performance on the task of maize tassel counting.

### Experiment on the WED

**Fig. 9.**
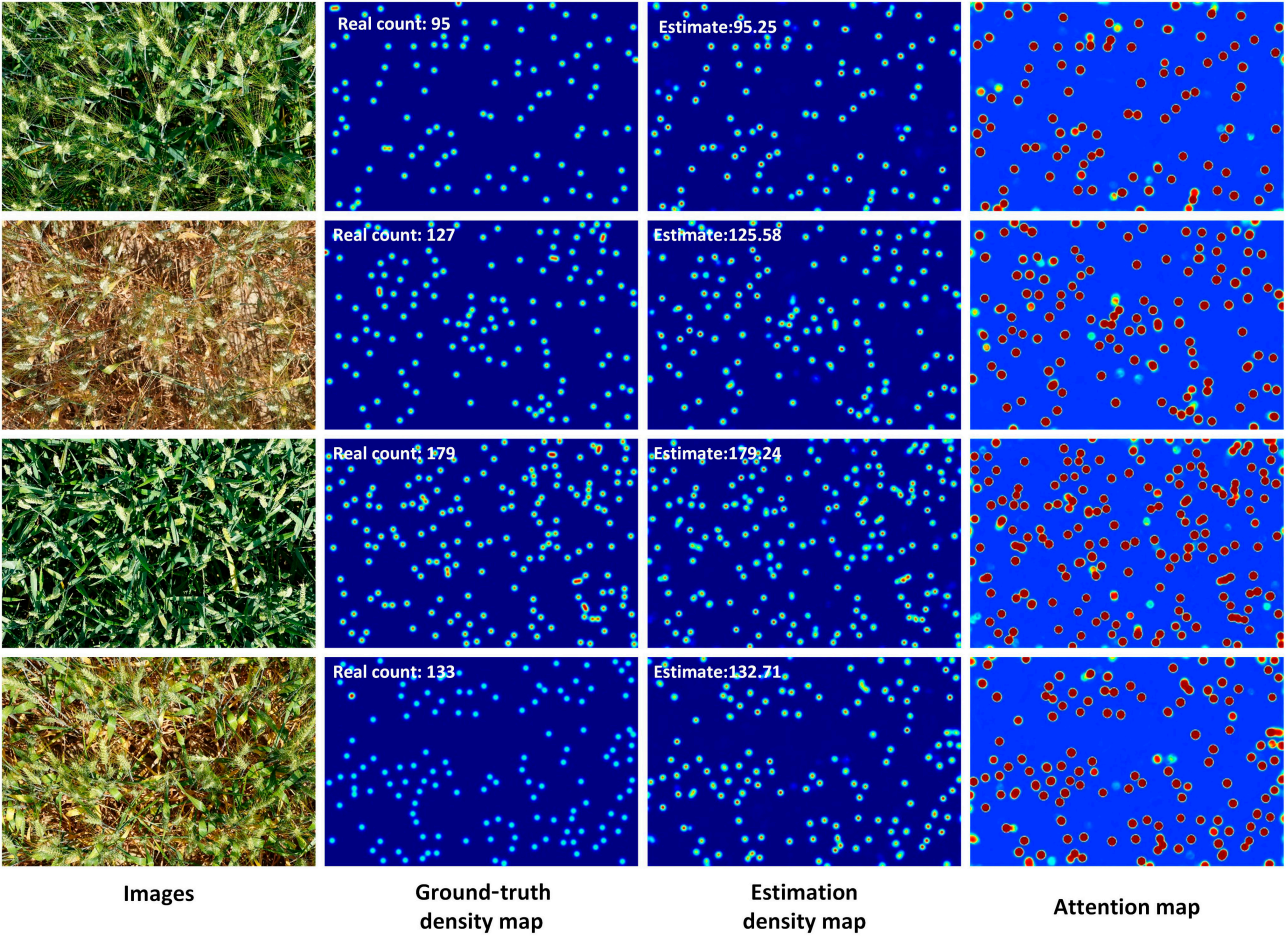
Visualization of the counting results in the WED dataset. The first column is the 4 test images, the second column is the ground-truth density maps, the third column is the estimated density maps, and the fourth column is the PAMs.

The WED is a widely used wheat ear dataset, which is first introduced by Madec et al. [6]. The wheat images collected in fields have 20 different genotypes. The image resolution is 6,000 × 4,000. The number of ears in each image varies from 80 to 170. This dataset includes 236 images, where 165 and 71 images are used for training and testing, respectively. For some genotypes, the color of wheat ears is extremely similar to the color of adjacent leaves. Bounding box annotations are provided in this dataset, while we only use the center point of each box in the experiment so as to unify the comparison of different methods. The resolution of the image is sampled to 1/8 of the original resolution and ϒ is set to 0.01. Image block in 256 × 256 was randomly cropped from those downsampled images and applied in the training. Other training details are consistent with the URC dataset. Table 3 shows the performance of different methods on the WED. The MAE and RMSE of our method reached 3.7 and 4.6, respectively. In particular, compared to the MCNN, we get 67.8% MAE and 70.5% RMSE improvement. We believe that the plant attention mechanism strengthens the ability of the proposed network to distinguish objects form image backgrounds and improves the counting accuracy. Moreover, we bring some counting results in the WED in Fig. 9. The results in Fig. 9 demonstrate that the network estimated density maps and counting results have a very high accuracy even when the distribution of the crop objects(wheat ears in the WED) is disordered. Table 3 and Fig. 9 indicate the well performance of the proposed RiceNet on the task of wheat ear counting.

**Table 3.** Performance of different methods on the WED.

**Networks**	**Venue, year**	**MAE**	**RMSE**
MCNN [30]	CVPR, 2016	11.5	15.6
CSRNet [17]	CVPR, 2018	4.2	5.2
BCNet [46]	TCSVT, 2019	4.1	4.9
Faster R-CNN [6]	AGRFORMET, 2019	4.6	5.9
SFC^2^Net [5]	PLPH, 2020	4.2	5.1
TasselNet [16]	PLME, 2017	6.8	8.3
TasslNetV2 [41]	PLME, 2019	5.3	6.8
**RiceNet**	**This paper**	**3.7**	**4.6**

### Coefficient of determination analysis of counting results

**Fig. 10.**
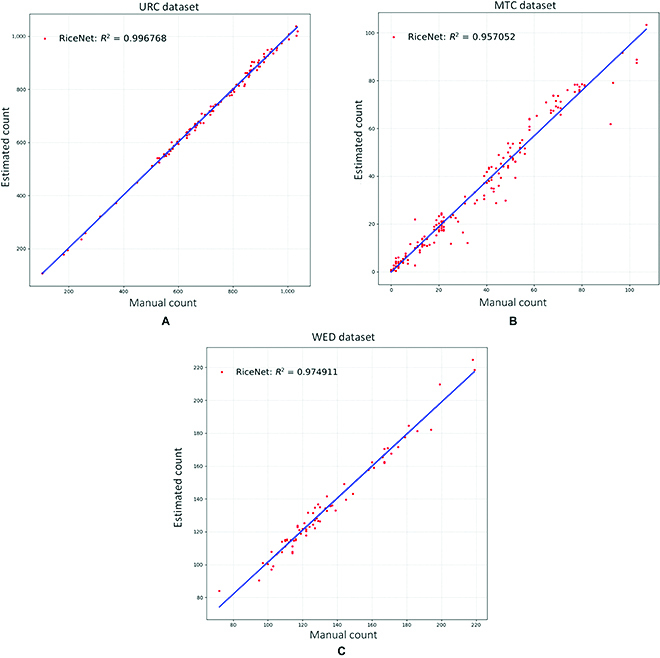
Coefficients of determination of the RiceNet on different datasets. (A), (B) and (C) are RiceNet counting results on URC, MTC and WED, respectively.

For the count results of the 3 different crop count datasets, we calculated their *R*^2^ values by Eq. 18. As shown in Fig. 10, the *R*^2^ values of 3 datasets reach 0.997, 0.957, and 0.975, respectively. Figure 10 demonstrates that the estimated counting results and the real values obtained by manual counting method have a high correlation. From another side, Fig. 10 indicates that our network not only has high counting accuracy but also has good generalization capabilities. According to the plots of coefficients of determination in Fig. 10, there are still some count results of being overestimated or underestimated, especially for the MTC dataset. This phenomenon is mainly due to the more severe shape and color variation of maize tassels in the MTC dataset.

$$R^{2}=1-\frac{\sum_{i=1}^{N}{\left(P_{i}-G_{i}\right)}^{2}}{\sum_{i=1}^{N}{\left(P_{i}-\bar{G}\right)}^{2}}$$ (18)

### Ablation experiment

In this section, we verify the effectiveness of the added positive–negative loss with the URC dataset. As shown in the last row of Table 4, the positive–negative loss can improve the counting performance of the proposed RiceNet on the URC dataset. Meanwhile, we incorporate positive–negative loss on other counting methods so as to explore the effectiveness of this loss function on other networks. Table 4 shows the effect of positive–negative loss in different models on the URC dataset. It can be seen from Table 4 that the performance of MCNN, CSRNet, and TasselNetV2 is improved after adding positive–negative loss. Compared with MCNN, MCNN with positive–negative loss decreases by 54.1% in MAE.

**Table 4.** The effect of positive–negative loss in different models on URC dataset.

**Networks**	**Without positive–negative loss**		**With positive–negative loss**	
	**MAE**	**RMSE**	**MAE**	**RMSE**
MCNN [30]	25.5	34.1	11.7↓	16.5↓
SANet [32]	10.1	13.4	10.8	14.9
CSRNet [17]	12.9	17.5	9.2↓	14.0↓
TasselNetV2 [41]	29.5	39.4	15.1↓	20.3↓
RiceNet	10.6	13.6	8.6↓	11.1↓

In addition, we analyze the influence of the parameter *σ* mentioned in Density map estimator section. The *σ* parameter determines the size of the Gaussian kernel when generating the ground-truth density map. From Table 5, we can see that when the *σ* value is 6 and the network performs best. We can observe from the Table 5 that the performances with different *σ* values do not change seriously. Comparable performance was achieved with Gaussian kernel sizes of 2 and 4. The experimental results in Table 5 show the robustness of our method to the Gaussian kernel size parameter.

**Table 5.** The effect of sigma in the generation of real density map.

*σ*	**MAE**	**RMSE**
2	8.7	11.5
4	8.8	12.1
6	8.6	11.2
8	9.2	12.5

### Estimation of plant location and size

In previous methods, to estimate the plant location and size, it is often necessary to resort to the bounding box labeling method. For rice UAV images with so many rice plants, bounding box labeling will be very time-consuming and labor-intensive, and it is not feasible. In contrast, the point labeling method is relatively simple, and it is easier to achieve a large number of labeling of the plants. In our method, on the basis of the reasonable assumption that rice plants are evenly distributed in each URC image, we utilize the distance between manual point annotations to generate pseudo-sizes as a supervision during network training. Thus, we achieve location and size estimation of rice plants with the manual point annotations and point-level supervisory. As seen from the fifth column of Fig. 7, with the designed PLD and PSE modules, RiceNet not only outputs the number of plants but also gives the location (red points) and size (white boxes) information of the rice plants. These location and size information are of great significance for subsequent crop phenotype researches and refined field managements. Since MTC and WED datasets are not satisfied with uniform distribution assumption, their location and size information cannot be calculated by the current version of RiceNet.

### Further discussions

In this section, we will further discuss the suggestions in the application of our rice plant counting, locating, and sizing network, the current shortcomings, and the future improvement directions. According to our three years (from 2018 to 2020) of experience in UAV image collection in paddy field and the after research of deep learning network design, we bring the following suggestions in the application of our RiceNet:

1. It is suggested not to collect UAV images on rainy days. Rainwater will not only cause damage to drone motors and gimbal camera but also cause image quality degradation and make the plant images to blur.
2. It is best to collect UAV images below the fifth-level wind speed. Excessive wind speed in field can enlarge the jitter of the drone resulting in an inaccurate camera autofocus. Moreover, it will change the shape of rice leaves and plants in rice image, giving rise to the decrease in network performance.
3. It is recommended to collect drone images in about 4 hours after sunrise, so as to avoid most fog time in the morning and the rice leaf curls caused by the high temperature at noon.
4. In our research, observers need to come the paddy field and perform drone control in each image collection mission. With the development of drone 5G technology, future 5G + UAVs can achieve remote flight control. In the future, the adoption of this type of UAVs can greatly reduce the difficulty of UAV image collection.
5. Following the usual field management methods, we used an appropriate amount of herbicide in the rice field. The appearance of some weeds (such as Barnyard grass, Scirpus juncoides, etc.) is very similar to the rice plants in paddy field. Without using herbicide, our network may degrade the counting performance because of the emergence of weeds.
6. It is recommended to use camera overhead imaging. Oblique imaging will cause occlusion between adjacent rice plants in the obtained image. Moreover, oblique imaging will add challenges to the estimation of plant location and size.

Next, we listed the shortcomings in our current RiceNet counting method and the possible research directions in the future:

1. During the periods of rice irrigation, sun reflection on the water may cause an overexposure region in the obtained image, as shown the last row of Fig. 7. The color information of the pixels in these overexposure region will be severely or completely lost. In future research, the design of new networks that can be more robust to sun reflection in the field will be an important research direction.
2. In RiceNet, multiscale feature fusion, plant attention mechanism, and positive–negative loss are adopted to suppress false positives from the image background. However, false positives still cannot be completely eliminated in the experiment. Therefore, the suppression of false positives from the background is still worth further research.
3. In this paper, the network performance of rice plant locating and sizing is qualitatively checked and evaluated with human eyes, since the URC dataset adopted plant point labeling instead of bounding box labeling. In future research, finding how to quantitatively evaluate the network performance of plant locating and sizing will be another important research direction.
4. In our project, we only need to focus on improving the accuracy of rice plant counting. All calculations were completed on a GPU-accelerated server after each UAV image collection mission (once every three days). Thus, we do not need to consider about of calculation complexity and running time. In future research, the design of lightweight networks that can run on the field robots or other embedded platforms in real time may also be a good research direction.

## Conclusion

This paper proposes a new rice plant counting, locating, and sizing approach with UAV imagery in paddy field. RiceNet includes one multiscale front-end feature extractor and 3 feature decoder modules (DME, PSE, and PLD). Plant attention mechanism and positive–negative loss are presented and utilized in the DME to improve the network’s ability to distinguish backgrounds to generate high-quality density maps. On the basis of the designed PLD and PSE modules, RiceNet not only can output the count number of rice plants but also can realize the plant location and size estimation with the manual point annotations. To verify our method, we present a new URC dataset, which consists of 355 images and 257,793 manual point annotations. In the experiment, the MAE and RMSE of RiceNet on the URC dataset are 8.6 and 11.2, respectively, which significantly outperforms states-of-the-art methods. Furthermore, we did counting experiments on two other famous crop counting datasets, and our method outperforms other methods by a large margin. In ablation experiments, we demonstrated the effectiveness of the added positive–negative loss and analyzed the influence of the sigma parameter on the counting performance. From the experiments, the proposed RiceNet can realize accurate, contactless, and efficient counting of rice plants in paddy field with UAV imagery. Moreover, RiceNet can provide higher-level semantic information (plant location and size). Access to those information can contribute to other crop phenotyping researches and help advance the development of automated fertilizer applicators, sprayers, etc. Last, we discussed the suggestions in the application of RiceNet, the current shortcomings of RiceNet, and the possible research directions in the future.

## Data Availability

The data presented in this study are available on request from the corresponding author.
